# The effects of intermittent fasting for patients with multiple sclerosis (MS): a systematic review

**DOI:** 10.3389/fnut.2023.1328426

**Published:** 2024-01-17

**Authors:** Xiaoxiao Lin, Shuai Wang, Yue Gao

**Affiliations:** ^1^Department of Geriatrics, Affiliated Hangzhou First People’s Hospital, School of Medicine, Westlake University, Hangzhou, Zhejiang, China; ^2^Zhejiang Key Laboratory of Traditional Chinese Medicine for the Prevention and Treatment of Senile Chronic Diseases, Hangzhou, Zhejiang, China; ^3^Zhejiang University School of Medicine, Hangzhou, Zhejiang, China

**Keywords:** intermittent fasting (IF), multiple sclerosis (MS), effects, autoimmune disease, systematic review

## Abstract

Some studies have investigated the impact of intermittent fasting (IF) for patients with multiple sclerosis (MS). We aimed to conduct a comprehensive systematic review to analyze and summarize all clinical studies concerning the effects of IF on patients with MS. We conducted an exhaustive review of information available in the Embase, Cochrane, and PubMed databases up until 1 September 2023. All clinical research relating to the impacts of IF for patients with MS were included. In total, this systematic review encompassed 5 studies, which included four RCTs and one pilot study. Each study involved was assessed of high quality. The results from these studies demonstrate that IF protocols could potentially serve as an effective dietary strategy for managing symptoms and improving the quality of life in individuals afflicted with MS. In conclusion, IF might be a potential beneficial dietary intervention for MS. However, the number of trials in this field is relatively limited. The large-scale clinical trials to investigate the effects of IF for MS are urgently needed, which may be helpful to manage this intricate neuroimmune disorder.

**Systematic review registration:**

https://inplasy.com, identifier INPLASY2023100021.

## Introduction

Multiple sclerosis (MS) is an autoimmune disease characterized by and neurodegeneration, demyelination, and chronic inflammation in the central nervous system (1–9). This complex disease manifests through a diverse range of neurological symptoms, often leading to severe physical and cognitive impairment. Current evidence suggests an interplay between genetic susceptibility and environmental factors, potentially triggering aberrant immune responses that result in CNS autoimmune aggression (10–12). The pathological hallmark of MS involves the autoimmune-mediated attack on myelin, the protective sheath encasing nerve fibers, leading to disrupted neuronal signaling and subsequent accumulation of disability. Clinical presentations of MS can vary significantly, characterized by episodes of neurological dysfunction known as relapses, which may be followed by periods of remission. The disease progresses in several phenotypes, with varying courses and prognoses. Despite extensive research, no cure for MS is available. Some ongoing research aims to unravel the underlying mechanisms of MS and to foster the development of more effective and targeted therapeutic strategies (13, 14).

The influence of dietary practices on the management and progression of MS holds significant interest (15–29), and emergent research indicates that certain dietary interventions could have ancillary benefits in symptom management, overall health enhancement, and potentially, the deceleration of disease progression. Some specific dietary protocols, namely the ketogenic diet (30–34), low-fat diet (28), Swank diet (35), Mediterranean diet (36–38), and intermittent fasting, have garnered attention for their purported benefits in the MS population. Intermittent fasting (IF), in particular, has surged in popularity due to preliminary studies from spanning animal models to human trials, suggesting its prospective role in symptom alleviation and disease course modulation.

Intermittent fasting (IF) regimens are generally categorized into three primary types: time-restricted eating (TRE), which involves consuming daily caloric intake within a consistent period, typically 8 h; alternate-day fasting (ADF), characterized by a rhythmic alternation between fasting and feasting days; and the 5:2 method, consisting of five designated eating days interspersed with two non-consecutive fasting days each week (39–43). Razeghi et al. (44) conducted seminal work using experimental autoimmune encephalomyelitis (EAE) to evaluate the implications of IF protocols on disease progression. Their investigations revealed that intermittent fasting did not adversely affect the progression of the disease. Notably, the application of IF during the prodromal stages of EAE appeared to attenuate the severity of the disorder, promoting a salutary effect evidenced by enhanced remyelination in the spinal cord. This dietary intervention was associated with a decrease in the secretion of inflammatory cytokines IFN-γ and TNF-α, concurrent with an increase in the anti-inflammatory mediator IL-10. The findings suggest a potential therapeutic role for IF in moderating early-stage EAE severity without accompanying deleterious effects. Supporting these observations, additional studies indicated clinical improvement and increased myelination in EAE models following IF. In the realm of clinical research, preliminary studies have explored IF’s impact on patients with multiple sclerosis. Fitzgerald et al.’s work (45, 46), posited that IF might serve as a viable approach for weight management and could potentially confer psychological benefits for individuals with MS. More recently, some studies (46, 47) explored the practicality and acceptability of TRE for adults with MS. Despite these investigative forays, a comprehensive systematic review synthesizing these clinical trials remains absent. Consequently, we undertake a systematic review to aggregate, scrutinize, and interpret the existing clinical investigations, aiming to elucidate the potential therapeutic efficacy and implications of IF strategies in the comprehensive management of MS.

## Materials and methods

### Search strategy

This systematic review was executed in strict adherence to the standards set forth by the Cochrane Collaboration. The review protocol, registered with INPLASY (number: 2023100021), and our reporting aligns with the Preferred Reporting Items for Systematic Reviews and Meta-Analyses (PRISMA) guidelines (47). A methodical search was conducted across three medical databases: Embase, PubMed, and the Cochrane Library, covering publications from their establishment to 1 September 2023. The search strategy encompassed a wide range of terminologies to capture relevant studies, employing both MeSH terms and free-text terms to ensure a comprehensive result. The terms included were variations and synonyms related to MS and IF, including specific dietary patterns within IF such as TRE, the 5:2 diet, and ADF. The following search terms were used: (“Multiple sclerosis” OR “Disseminated Sclerosis” OR MS OR “Multiple Sclerosis, Relapsing-Remitting” OR “Multiple Sclerosis, Acute Relapsing” OR “Multiple Sclerosis, Relapsing Remitting” OR “Multiple Sclerosis, Remitting-Relapsing” OR “Relapsing Remitting Multiple Sclerosis” OR “Relapsing-Remitting Multiple Sclerosis” OR “Remitting Relapsing Multiple Sclerosis” OR “Remitting-Relapsing Multiple Sclerosis”) AND (“intermittent fasting” OR time-restricted OR TRE OR “time-restricted eating” OR “time-restricted feeding” OR TRF OR “5:2 diet” OR “Alternative-day fasting” OR “Alternative day fasting” OR Alternative-day OR ADF OR “intermittent calorie restriction.”) After the electronic database search, we conducted an additional manual search to scrutinize the bibliographies of pertinent reviews and studies. The search process and study selection were undertaken by two independent researchers (XL and SW). Both authors embarked on the initial screening based on titles and abstracts, followed by a full-text review of shortlisted studies. In instances of ambiguity or disagreement regarding study inclusion, a third author (YG) was consulted to reach a consensus, ensuring the objectivity and integrity of the selection process.

### Inclusion criteria

Utilizing the PICOS (Population, Intervention, Comparison, Outcomes, and Study Types) structure, we defined our inclusion criteria for this systematic review as follows: Population: The study targeted individuals diagnosed with MS, irrespective of disease stage or demographic characteristics. This broad criterion was intended to capture a diverse array of participants, thereby providing a comprehensive overview of intermittent fasting’s applicability across different MS subgroups. Intervention: We focused on studies that employed intermittent fasting strategies, specifically those incorporating ADF, the 5:2 dietary regimen, or TRE. These interventions represent varied approaches within the spectrum of intermittent fasting, thus facilitating a nuanced analysis of different fasting protocols. Comparison: The comparison group comprised individuals with MS who were not subjected to any form of intermittent fasting, allowing for a clear contrast between standard dietary patterns and those of the intermittent fasting interventions. Outcomes: The primary outcomes of interest were the feasibility, acceptability, efficacy, and safety of intermittent fasting protocols within the MS population. These multidimensional endpoints were chosen to evaluate not only the clinical and health implications but also the practicality and tolerability of adopting such dietary modifications in everyday living scenarios for individuals with MS. Study Types: We included a wide range of empirical studies to ensure methodological rigor and comprehensive data synthesis. Eligible studies encompassed cross-sectional analyses, case-control studies, cohort studies, and randomized controlled trials (RCTs). To maintain the focus and quality of the data, we excluded editorials, duplicates, commentaries, conference abstracts, supplements, and case reports.

### Quality appraisal and data extraction

In the meticulous process of appraising the methodological rigor of included studies, our review employed specific tools tailored for diverse study designs. For RCTs, the RoB 2.0 tool was utilized (48), a robust instrument endorsed by the Cochrane Collaboration for its precision in evaluating the risk of bias across various domains within a study. These critical domains encompass the randomization process, deviations from intended interventions, discrepancies in outcome data, outcome measurement fidelity, and the selectivity in reported results. For non-randomized studies of interventions (NRSIs) we applied the ROBINS-I tool (49), a specialized instrument adept in dissecting the risk of bias in studies outside the randomized controlled paradigm. This tool systematically scrutinizes seven domains prone to bias, including confounding elements, selection of participants, classification of interventions, deviations from intended interventions, data incompleteness, outcome measurement, and reporting selectivity. In conducting the quality assessment, a scoring system was implemented to quantify risk: 2 points were allocated for low risk, 1 point for unclear risk, and 0 points were assigned for high risk. To synthesize the outcomes of these assessments, we categorized the accumulated scores into three distinct tiers of research quality: low, moderate, and high. Accordingly, studies were stratified based on their total points, with scores between 0 and 4 was classified as “low quality.” Those accumulating 5 to 9 points fell into the “moderate quality” bracket, while a score ranging from 10 to 14 signified “high quality.” The intricate process of data extraction was independently undertaken by two authors (Lin and Wang), ensuring objectivity and consistency. We meticulously organized the extracted data into structured tables for enhanced clarity and accessibility in analysis. The initial table encapsulated comprehensive study descriptors, encompassing an array of characteristics from publication details to participant demographics and methodological particulars. A subsequent, dedicated table succinctly articulated the primary outcomes, complemented by results from our rigorous quality assessments.

## Results

### Literature search

**FIGURE 1:**
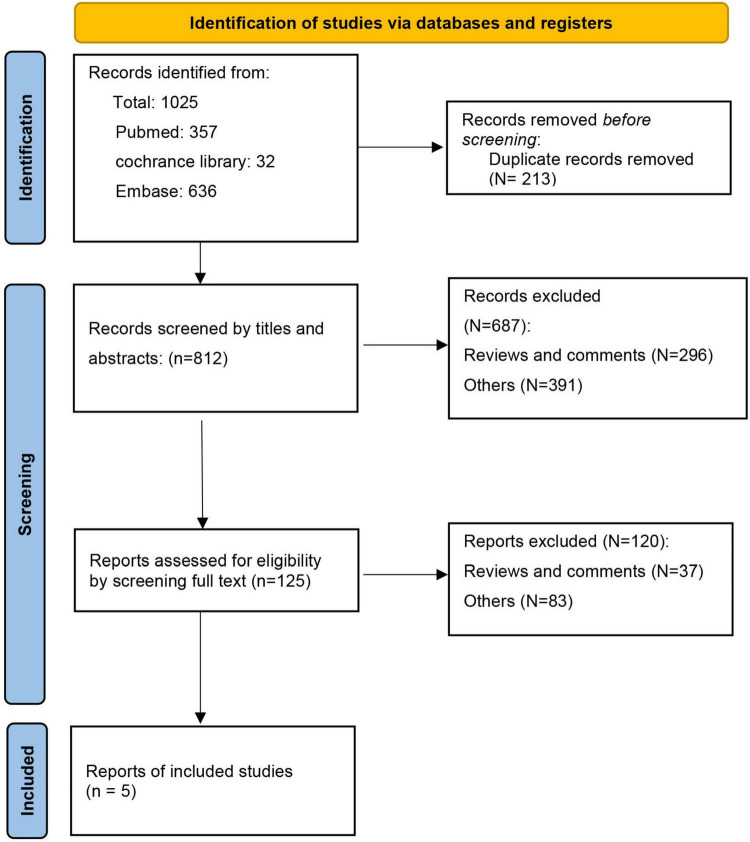
Search flow diagram.

The initial exploration of relevant databases resulted in 1,025 potential records. Upon executing a meticulous process to eliminate duplicates, we streamlined the list to 812 unique records. These records were then subjected to a rigorous screening process, which involved a review of the titles, abstracts, and full texts to ascertain their relevance and adherence to our predefined criteria. This stringent selection process culminated in the identification of five studies that were deemed appropriate for inclusion in our systematic review (45, 46, 50–52). Of these, four were RCTs and one was a pilot study. The methodical progression of the search and study selection procedures is visually represented in Figure 1.

### Study and patient characteristics, and the assessment of quality

**FIGURE 2:**
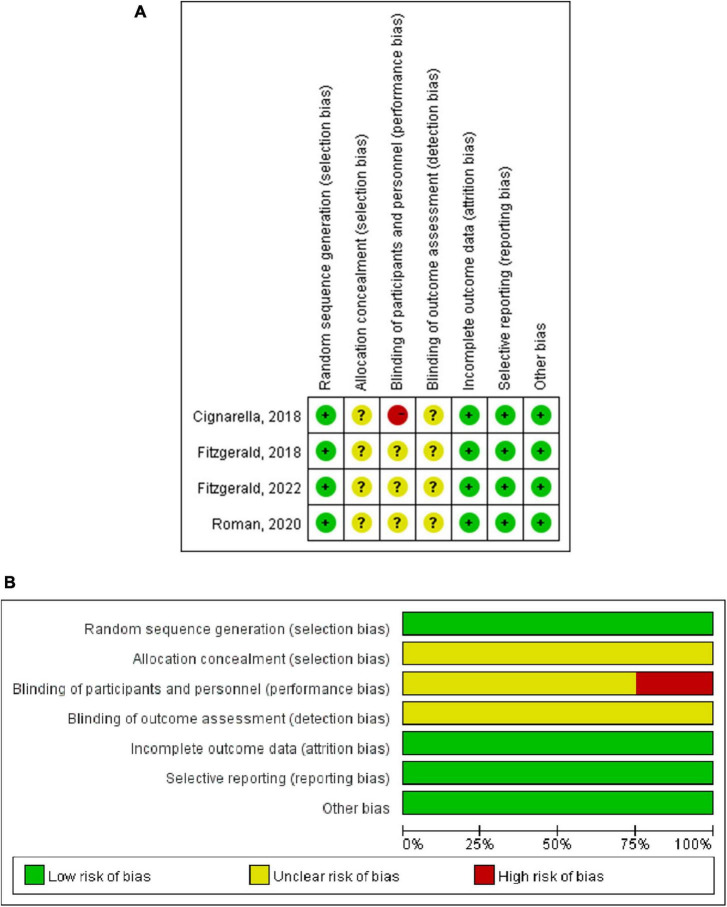
Risk of bias of RCTs by the Cochrane risk assessment tool. **(A)** Risk of bias graph. **(B)** Risk of bias summary.

Within the studies incorporated in this systematic review, the sample sizes of individual investigations displayed variability, encompassing a spectrum from 12 to 70 participants. Participant demographics indicated a mean age extending from approximately 37.4 to 46 years. The proportion of female participants in these studies was notably high, oscillating between 75 and 82%. Regarding dietary protocols, the studies were categorized based on the type of IF regimen implemented. One study explored the ramifications of ADF, while three studies investigated the 5:2 diet and two explored the TRE. These interventions exhibited a duration ranging from a minimum of 15 days to a maximum of 56 days. The study characteristics were summarized in Table 1. For the quality assessment of these studies, four studies were assigned scores of 10 and 11 via the RoB2 tool and one study assessed through the ROBINS-I tool, attained a score of 12. All studies were evaluated as high quality, which was shown in Figure 2 and Table 2.

**TABLE 1:** A comprehensive summary of study characteristics.

References	Design	The sample size	Female, %	Mean age	Intervention group	Control group	Intervention time	Main measured outcomes
(50)	RCT	16	75%	41.0 years	ADF	Libitum control diet	15 days	Serum adipokines and other metabolites, peripheral blood leukocytes, and gut microbiome.
(46)	RCT	36	80.60%	37.4 years	5:2 diet	Daily CR diet and weight-stable diet	28 days	The safety and feasibility, changes in weight, fasting glucose, lipid levels, and changes in PROs including fatigue, sleep and mood.
(45)	RCT	36	80.60%	37.4 years	5:2 diet	Daily CR diet and weight-stable diet	28 days	The changes in cryopreserved peripheral blood mononuclear cells.
(51)	RCT	70	77.60%	40.1 years	TRE and 5:2 diet	Libitum control diet and daily CR diet	56 days	The feasibility, change in weight/BMI, PROs of fatigue, sleep quality, quality of life.
(52)	Pilot study	12	82%	46 years	TRE	NA	56 days	The feasibility and acceptability, MS outcomes, PROs, anthropometrics, Body composition.

**TABLE 2:** Main findings and the quality assessments.

References	Main findings	The score of quality assessment
(50)	IF altered blood adipokines and the gut flora resembling protective changes observed in mice. In conclusion, IF has potent immunomodulatory effects that are at least partially mediated by the gut microbiome.	10
(46)	IF may be an effective diet for weight loss in patients with MS, and may be associated with improved emotional health.	11
(45)	In people with MS, an intermittent CR diet was associated with reduction in memory T cell subsets and certain biologically-relevant lipid markers.	11
(51)	Diet adherence remains a primary barrier to the feasible conduct of large, randomized controlled diet trials. Strict adherence to a TRF dietary change may be more feasible than calorie restriction and should be considered in future IF trials.	11
(52)	TRE may be feasible and acceptable in adults with MS. Further, participants reported qualitative improvements in fatigue, sleep, and wellbeing.	12

### The effects of IF for patients with MS

In a preliminary randomized controlled trial conducted by Cignarella et al. (50), the impact of IF on patients experiencing a relapse of MS was explored, focusing particularly on short-term metabolic shifts and gut microbiome alterations. The small cohort, comprising 16 individuals diagnosed with MS, yielded intriguing results. Notably, there was a marked reduction in leptin levels across both the IF cohort and the control group, though the decline was more pronounced in the former. Similarly, both groups experienced an upsurge in adiponectin levels, albeit without significant intergroup disparities. A critical immunological observation was the contrasting lymphocyte dynamics between the groups; while absolute T and B lymphocyte counts escalated in the control group, these either diminished or plateaued in the IF group. Moreover, an assessment of microbiome changes revealed an upward trend in the populations of *Faecalibacterium*, *Lachnospiraceae incertae sedis*, and *Blautia* within a 15-day span of intermittent fasting; however, these variations did not significantly diverge between the groups. Based on these findings, the researchers posited that IF potentially augments gut microbial diversity and modifies both the compositional structure and metabolic pathways, implications that might herald therapeutic advantages for individuals grappling with MS. This pilot study, therefore, underscores the prospective role of dietary interventions in modulating physiological responses during MS relapses, although further expansive studies are warranted to substantiate these initial findings.

In an explorative randomized controlled feeding study spearheaded by Fitzgerald et al. (46), the safety and practicability of various calorie restriction (CR) diets were evaluated, and a sample of 36 participants with MS were included. The study revealed a more pronounced weight reduction in the cohort following traditional calorie restriction as compared to its IF counterparts. It was noted, however, that adherence levels were comparatively lower in the IF group than in those undergoing consistent calorie restriction. Both the CR and IF regimens were associated with significant enhancements in emotional wellbeing, underscored by marked improvements in depression scores amongst participants. These findings led the researchers to conclude that intermittent fasting emerges as a safe and viable approach to weight loss in individuals afflicted with MS. Furthermore, the regimen may carry adjunctive benefits relating to emotional health, a consideration of substantial potential relevance in the holistic management of MS. In subsequence analysis, Fitzgerald et al. (45) explored the effects of different CR diets over 8 weeks. Participants were grouped into three diets: a control diet with consistent calorie intake, a daily CR diet with 78% of daily calorie needs, and the 5:2 diet. Of the participants, 86% completed the trial. Those on the intermittent CR diet displayed significant reductions in memory T cell subsets, especially effector memory and Th1 cells, and an increase in naïve T cell subsets. This change was not seen in the daily CR or control groups. Additionally, those on the IF diet who showed major changes in certain lipid markers also displayed notable changes in T cell subsets. The study concludes that intermittent CR might benefit MS patients by reducing specific T cell subsets and influencing lipid markers.

Roman et al. (51) conducted an analysis of three pilot studies were conducted, focusing on either restricting calorie intake daily or intermittently, or TRE. Of the 90 participants, 70 completed the study without any serious side effects. The three studies conducted between 2015 and 2017 at the Johns Hopkins MS Center explored the impacts of various calorie restriction diets on individuals with relapsing-remitting multiple sclerosis (RRMS). Study 1 (ATAC-MS) found that most participants struggled with long-term adherence to a self-directed intermittent calorie restriction diet, with minimal sustained benefits in weight loss or patient-reported outcomes (PROs) post the initial 8-week phase. Study 2 revealed that allowing participants to choose their calorie restriction method did not significantly enhance long-term adherence or PROs, regardless of additional communication support. Participants who did adhere and lost weight noted an improvement in fatigue. Study 3 assessed the feasibility of a TRE diet and found higher adherence rates, with most participants still following the diet by study’s end. However, there were no significant changes in weight or PROs over the study period. Across all studies, sustained dietary adherence was a challenge, and noticeable benefits were minimal, underscoring the need for more engaging, sustainable strategies and exploring further how these dietary approaches may influence MS symptoms and overall patient wellbeing. The study evaluated the safety and feasibility of various forms of calorie and TRE among individuals with MS. While the diets were generally well-tolerated, adherence was low despite different levels of support, indicating that self-directed dietary interventions are challenging for this population. No significant change was observed in weight or patient-reported outcomes, possibly due to low adherence or other factors like drop-out bias. However, there were trends suggesting potential benefits, consistent with earlier studies showing dietary modifications could improve quality of life aspects in MS. The research underscores the necessity for larger, more controlled studies to conclusively determine the clinical benefits of these diets in managing MS, highlighting the need for effective strategies to enhance adherence and exploring modern technologies’ roles in support and measurement. Further, TRE emerged as a promising focus for future investigations on diet’s impact on MS outcomes.

Wingo et al. (52) conducted a pilot study to investigate the feasibility and acceptability of a TRE protocol among adults with RRMS. Throughout the 8-week trial, participants (*n* = 12) were required to consume their daily food intake within an 8-h window and fast for the remaining 16 h. The study found a high retention rate and overall adherence, with participants maintaining the eating pattern on average > 6.5 days/week, and noted qualitative improvements in various aspects like sleep, fatigue, and overall wellbeing. Despite the limited sample size and lack of control group, which prevents causal interpretations, the results are promising, indicating potential benefits of TRE in managing MS symptoms, particularly cognitive functions and motor skills, as evident from improvements in specific tests. Given the positive feedback from participants and the low-cost, scalable nature of TRE, the study suggests further, more comprehensive trials to conclusively determine the intervention’s effectiveness in reducing MS symptoms, emphasizing the approach’s advantages in terms of flexibility and adherence.

## Discussion

To the best of our knowledge, this systematic review represents the first comprehensive synthesis and analysis of clinical trials examining the efficacy of IF as a dietary intervention in patients with MS. Through rigorous criteria, our search and analytical methodology yielded five pertinent studies. The cumulative evidence from these studies suggests that IF protocols could potentially serve as an effective dietary strategy for managing symptoms and improving the quality of life in individuals afflicted with MS. This assertion is predicated on the consistent patterns observed across the evaluated studies, underscoring the need for further large-scale, randomized controlled trials to validate these preliminary findings.

The included studies have investigated the impact of IF on MS symptoms, patient-reported quality of life, and physiological indicators pertinent to the disease. One groundbreaking study by Cignarella et al. (50) highlighted the transformative effects of IF on metabolic parameters and gut microbiota composition. The research documented an increase in microbial diversity and a shift in the gut flora following a 15-day IF regimen, inferring a potential therapeutic impact of IF on auto-immune conditions through gut health modulation. This connection is particularly significant considering the burgeoning evidence correlating intestinal health with auto-immune disorders (53–55). In a parallel inquiry, Fitzgerald et al. (46) demonstrated that IF protocols extend beyond mere weight management benefits for individuals with MS, potentially fostering improvements in emotional health. Subsequent investigations (45) indicated that intermittent calorie restriction distinctly influences T cell populations, suggesting a role in immune response regulation—a vital aspect of MS management, given its foundational association with immune system aberrations. Nonetheless, the practicality of sustained adherence to restrictive CR diets poses a considerable challenge, as outlined by Roman et al. (51). The rigors of strict calorie limitation often deter continued compliance, underscoring the necessity for more accessible dietary interventions. Contrastingly, TRE emerges as a more user-friendly alternative, as documented by Wingo et al. (52)., offering substantive metabolic and physiological shifts akin to those prompted by more stringent CR methods, without the associated adherence hurdles. The comparative ease and significant outcomes of TRE could potentially translate into a higher patient adherence rate, thereby making it a more sustainable option for long-term dietary management in MS. The question of dietary adherence in therapeutic interventions, particularly within the context of IF diet trials, is paramount when considering patient-centric outcomes. One emerging consideration, based on recent evidence, is the potential feasibility of TRE over traditional CR methods (52). In summation, the landscape of dietary interventions for MS—each with its distinct merits and challenges—requires careful consideration, particularly regarding the sustainability of long-term adherence. Future studies would expand on the existing knowledge base, exploring the mechanistic pathways through which these dietary protocols impact MS. Recent scientific discourse has increasingly centered on the potential therapeutic implications of IF in the management of MS and other autoimmune maladies (56, 57). Current research suggests several mechanisms through which IF may exert neuroprotective effects for patients with MS (56, 57), including: (1) regulate blood glucose level and enhance insulin sensitivity. (2) Suppress inflammatory response (3) Autophagy Activation. (4) Reduce oxidative stress.

Our study acknowledges several inherent limitations that necessitate a cautious interpretation of the findings. Primarily, the scope of research examining IF within the context of MS patients remains notably restricted, evidenced by the mere five clinical studies identified that directly pertain to this investigative domain. This scarcity of studies is further compounded by the second limitation: the relatively diminutive sample sizes within the existing clinical trials. Such constrained participant numbers may potentially limit the generalizability of the findings, representing a significant hurdle in unequivocally substantiating the therapeutic efficacy of TRE in managing MS symptoms and disease progression. Given these constraints, there is an exigent need for additional research. Future studies, ideally with larger and more diverse cohorts, are imperative to elucidate the comprehensive impact and potential benefits of TRE in the MS population. Expanding the breadth of research in this area will not only affirm the findings from preliminary studies but also enhance our understanding of the intricate mechanisms by which dietary interventions may influence disease trajectories in autoimmune conditions such as MS.

In conclusion, IF might be a potential beneficial dietary intervention for MS. However, the number of trials in these fields is relatively limited. Preliminary studies have hinted at the beneficial effects of IF in managing MS; however, these initial assessments are derived from smaller cohorts. IF showed as a good diet pattern in weight-loss trials or improved quality of life due to its ability to induce caloric restriction and bring about metabolic changes, affect various hormones related to hunger and metabolism, such as improving insulin sensitivity, and give the digestive system a rest, potentially improving gut health, and may reduce inflammation, contributing to overall health benefits. The unconfirmed aspects of IF in clinical trials, particularly among patients with diseases originating from inflammation, include uncertainties about its long-term effects and specific impacts on inflammatory conditions. While some studies suggest that IF might reduce inflammation markers, comprehensive evidence on how it affects chronic inflammatory diseases, such as MS, is still limited. The variation in fasting protocols such as the duration and frequency of fasting periods makes it difficult to generalize results. Additionally, the potential impact of IF on the immune system, which is closely tied to inflammatory responses, is not fully understood. Consequently, there is a pressing need for large-scale clinical trials designed to rigorously investigate the therapeutic potential and mechanistic underpinnings of IF in the context of MS.

## Data availability statement

The original contributions presented in this study are included in this article/supplementary material, further inquiries can be directed to the corresponding authors.

## Author contributions

XL: Conceptualization, Data curation, Investigation, Methodology, Software, Writing – original draft. SW: Data curation, Formal Analysis, Methodology, Project administration, Supervision, Validation, Writing – original draft, Writing – review & editing. YG: Formal Analysis, Funding acquisition, Project administration, Resources, Visualization, Writing – original draft, Writing – review & editing.
